# GenNon-h: Generating multiple sequence alignments on nonhomogeneous phylogenetic trees

**DOI:** 10.1186/1471-2105-13-216

**Published:** 2012-08-28

**Authors:** Anna M Kedzierska, Marta Casanellas

**Affiliations:** 1 Centre for Genomic Regulation, Dr. Aiguader 88, 08003 Barcelona, Spain; 2 Departament de Matemàtica Aplicada I, ETSEIB, Universitat Politècnica de Catalunya, Avinguda Diagonal 647, 08028 Barcelona, Spain

## Abstract

**Background:**

A number of software packages are available to generate DNA multiple sequence alignments (MSAs) evolved under continuous-time Markov processes on phylogenetic trees. On the other hand, methods of simulating the DNA MSA directly from the transition matrices do not exist. Moreover, existing software restricts to the time-reversible models and it is not optimized to generate nonhomogeneous data (i.e. placing distinct substitution rates at different lineages).

**Results:**

We present the first package designed to generate MSAs evolving under discrete-time Markov processes on phylogenetic trees, directly from probability substitution matrices. Based on the input model and a phylogenetic tree in the Newick format (with branch lengths measured as the expected number of substitutions per site), the algorithm produces DNA alignments of desired length. GenNon-h is publicly available for download.

**Conclusion:**

The software presented here is an efficient tool to generate DNA MSAs on a given phylogenetic tree. GenNon-h provides the user with the nonstationary or nonhomogeneous phylogenetic data that is well suited for testing complex biological hypotheses, exploring the limits of the reconstruction algorithms and their robustness to such models.

## Background

The package GenNon-h presented here simulates DNA sequences evolving on a given phylogenetic tree directly from the transition matrices. In other words, the goal of developing GenNon-h was to generate DNA sequences following *discrete-time* Markov models on a given phylogenetic tree (with assigned branch lengths measured as the expected number of substitutions per site). Given a phylogenetic tree *T*, the parameters of these models are the root distribution and the substitution matrices *P*_*e*_ assigned to each edge *e* of *T*. The entries of *P*_*e*_ correspond to the conditional probabilities *P*(*x*|*ye*) that a nucleotide *y* at the parent node of *e* is substituted by nucleotide *x* at the child node (we refer to [1, chapter 8] for an introduction to these type of Markov processes). There is no extra restriction on these matrices, in contrast to *continuous-time* processes, where the substitution matrices are the exponential of a rate matrix *Q* multiplied by the number of events. Whereas generating matrices of type *exp*(*tQ*) with a preassigned number of substitutions (branch length) is relatively easy, the task of generating (discrete-time) Markov matrices with a preassigned expected number of substitutions per site has been recently solved in [2]. The software GenNon-h is implemented for the (discrete-time) Jukes-Cantor JC69, Kimura two K80 and three K81 parameters, strand symmetric SSM, and general Markov models GMM.

The shape of the substitution matrices (in all cases sum of rows is equal to 1 and the entries are nonnegative) for JC69, K80, K81, SSM and GMM models is given by:

$$\left(\begin{array}{llll} a & b & b & b\\ b & a & b & b\\ b & b & a & b\\ b & b & b & a \end{array}\right)\;\left(\begin{array}{llll} a & b & c & b\\ b & a & b & c\\ c & b & a & b\\ b & c & b & a \end{array}\right)\;\left(\begin{array}{llll} a & b & c & d\\ b & a & d & c\\ c & d & a & b\\ d & c & b & a \end{array}\right)$$

$$\left(\begin{array}{llll} a & b & c & d\\ e & f & g & h\\ h & g & f & e\\ d & c & b & a \end{array}\right)\;\left(\begin{array}{llll} a & b & c & d\\ e & f & g & h\\ i & j & k & l\\ m & n & o & p \end{array}\right).$$

For these models, the algorithms given in [2] can generate any stochastic matrix corresponding to a given branch length (except for the general Markov model, where the algorithms cover a dense set of matrices that is not proved to be complete). We refer the reader to [2] for more details and references of these models.

In the continuous-time models, the process is often assumed to be *homogeneous* (with the same rates of substitutions in all lineages) and the rate matrix *Q* is common throughout the entire tree. This is not an implicit hypothesis for the discrete-time Markov processes, thus the evolutionary processes simulated using GenNon-h are nonhomogeneous. Although a variety of methods exist for simulating alignments under the continuous-time models (see for example Rose[3], Seq-Gen[4], and Evolver in PAML[5]), we provide the first software for generating multiple sequence alignments evolving under the discrete-time Markov processes on trees.

Other powerful software include the Bayesian phylogenetic methods of [6] and [7]. MRBAYES[6] is a robust inference and model selection method, which provides a variety of tools for nucleotide and amino-acid data analysis. BEAST[7] enables to generate MSA with rate matrices differing at distinct edges. However, both packages are based on the rate-based continuous-time models for nucleotide data and as such, assume the exponential form of the substitution matrices. This in turn restricts the space of possible matrices assigned to a given edge length. GenNon-h, which aims to explore the space of all matrices, mimics the stochastic character of evolution as attempted to be captured by mathematical models. GenNon-h is the first software tailored specifically for the purpose of generating the DNA sequence alignments evolving on phylogenetic trees under the nonhomogeneous models. GenNon-h does not aim to replace the existing methods, but to serve as an option for researchers, whose interest lies in testing the performance of the algorithms on data generated under the assumption of more general models. A newly created software Empar ( http://genome.crg.es/cgi-bin/phylo_mod_sel/Empar.pl, [8]), enables to infer the parameters of the model considered in this work.

It is worth pointing out that the strand symmetric model and the general Markov model considered in GenNon-h do not lie in the class of stationary models, which adds to the flexibility of the framework presented here. As a comparison, we note that the software packages that are prevalently in use consider only stationary and time-reversible models. This is due to the fact that the continuous-time models require computing exponentials of rate matrices, which is a nontrivial task for a general matrix. This contributes to the practice of using the time-reversible rate-based models irrespective of the setting under consideration.

As shown in [9], the substitution parameters for the GMM model (and thus for all its submodels), are identifiable up to permutation. In GenNon-h we fabricate matrices of the *Diagonal Largest in Column* (*DLC*) type [10], whenever possible, i.e. matrices whose largest entry in every column is placed on the diagonal. *DLC* matrices share an important feature of being identifiable– there exists a unique set of substitution matrices satisfying the *DLC* condition and a unique root distribution that leads to the given joint distribution at the leaves. In other words, the data generated from the *DLC* matrices and sufficient alignment lengths have high likelihood of being identifiable and therefore can be safely used to test hypotheses about the tree or the data.

## Implementation

GenNon-h has been implemented in C++. Its input is a tree in the Newick format (rooted or unrooted, with nodes of any degree) with annotated branch lengths. Other arguments include the base name of the output files, length of the alignment and a model. An exemplary command-line input is: 

 ./GenNon-h treefilename outputfilename length modelname

For instance, if ‘tree.txt’ is a text file consisting a Newick 5-taxon phylogenetic tree: 

 ((species1:0.01,species2:0.2,species3:0.3):0.5,species4:0.4,species5:0.7),

then the following command line input 

 ./GenNon-h tree.txt data.fa 10000 k81

generates a MSA of length 10,000nt evolving on the tree given in ‘ tree.txt’ under the K81 model. The result is recorded it in the file ‘data.fa’.

The algorithm proceeds as follows: 

Input: a discrete-time Markov model M, a phylogenetic tree T with assigned branch lengths in the Newick format, and an alignment length *L*;

Step 1: generate a DNA sequence *s*_0_of length *L* at the root of T according to distribution of the model M;

Step 2: for each edge *e* in T, using [2], we generate a matrix *P*_*e *_of the type M corresponding to the length *l*_*e *_of edge *e* (i.e. a matrix *P*_*e *_such that $l_{e}=-\frac{1}{4}\text{log}\text{det}(P_{e})$). If the resulting matrix is not *DLC*, we apply a permutation of rows to convert it to a *DLC* matrix. Every model has a set of permutations allowable, such that the form of the matrix dictated by the model is maintained. If neither of the permutations creates a DLC matrix, we generate a new matrix *P*_*e *_and repeat the procedure. We limited the number of trials to 1000 before the simulations require a re-start, however, in practice a *DLC* matrix is expected to be found in much fewer iterations;

Step 3: we let *s*_0_ evolve according to the corresponding Markov process on T;

Output: a multiple sequence alignment and the substitution matrices used for its simulation.

The output files constitute both a fasta file with a multiple sequence alignment simulated on T, and a file listing the parameters used for the simulations of the data. The order of the output matrices corresponds to the order in which the branches of the Newick tree are read: terminal edges are put first, followed by the top-down listing of the edges starting at the root (see package README for detailed information).

## Results and discussion

The C++ implementation of GenNon-h can be accessed at the project home page http://genome.crg.es/cgi-bin/phylo_mod_sel/AlgGenNonH.pl, at Sourceforge, or in the Additional file 1 accompanying this paper. The algorithm has been successfully used in [11] to test a new model selection method based on 4 and 6-taxon DNA multiple sequence alignments. The size of the tree is not a limiting factor to the use of the code as the execution time grows linearly in the number of taxa, thus the method can be used to generate data of any size.

In order to test the speed of GenNon-h, we investigated the times it took to generate 100 alignments of 1,000nt on the following 5-taxa tree ((*seq*1:0.01,*seq*2:0.2,*seq*3:0.3):0.5,*seq*4:0.4,*seq*5:0.7). The results are given in Table 1.

**Table 1:** **GenNon-h****: time to generate 100 alignments of length 1,000bp on a 5-taxon tree on a Macintosh 2.4 GHz Intel Core 2 Duo with 4GB**

**Model**	JC69	K80	K81	SSM	GMM
Time	2.6s	2.6s	2.5s	2.6s	3.0s

The simulated data saved in the output files together with the parameters used for its simulation are suited for hypothesis testing in a variety of biological applications.

## Conclusions

GenNon-h is the first software simulating DNA sequences directly from the transition matrices computed from the branch lengths of a given phylogenetic tree. It implements discrete-time Markov processes on phylogenetic trees on any number of taxa. In the current release the support was given to the most well-known discrete-time Markov models: the Jukes-Cantor, Kimura 2 and 3 parameters, strand symmetric and general Markov models.

The possibility of generating nonhomogeneous (for any of the models above) or nonstationary (for the SSM or GMM models) processes makes the method particularly appealing when handling complex biological questions.

## Availability and requirements

**Project name**: GenNon-h

**Project home page**: http://genome.crg.es/cgi-bin/phylo_mod_sel/AlgGenNonH.pl; also available from SourceForge https://sourceforge.net/projects/gennonh/, and as Additional file 1

**Operating systems**: Platform independent

**Programming language**: C++

**Other requirements**: GNU gcc compiler, version 1.47.0 of the boost library ( http://www.boost.org/)

**Distributed under** the GNU General Public License

## Abbreviations

JC69: discrete-time Jukes-Cantor model; K80: discrete-time Kimura 2-parameter model; K81: discrete-time Kimura 3-parameter model; SSM: strand symmetric model; GMM: general Markov model.

## Competing interest

Both authors declare that they have no competing interests.

## Author’s contributions

AK created and tested the software, established a platform for its usage and drafted part of the manuscript. MC conceived of the project and drafted part of the manuscript. Both authors read and approved the final manuscript and declare no conflicts of interests.

## Supplementary Material

Additional file 1 **Is a zipped (extension .zip) file containing the C++ implementation of ****GenNon-h.** click here for file
